# Association of Phase Angle, but Not Inflammation and Overhydration, With Physical Function in Peritoneal Dialysis Patients

**DOI:** 10.3389/fnut.2021.686245

**Published:** 2021-05-31

**Authors:** Vanessa Mota Silva, Maryanne Zilli Canedo Silva, Barbara Perez Vogt, Nayrana Soares Carmo Reis, Fabiana Lourenço Costa, Mariana Souza Dorna, Marcos Ferreira Minicucci, Jacqueline Costa Teixeira Caramori

**Affiliations:** ^1^Clinical Hospital of Botucatu Medical School, Multiprofessional Specialization in Adult and Elderly Health, São Paulo State University, UNESP, Botucatu, Brazil; ^2^Internal Medicine Department, Botucatu Medical School, São Paulo State University, UNESP, Botucatu, Brazil; ^3^Graduate Program in Health Sciences, Medicine Faculty, Federal University of Uberlândia (UFU), Uberlândia, Brazil

**Keywords:** bioelectrical impedance, functional capacity, inflammation, phase angle, overhydration, short physical performance battery

## Abstract

**Introduction:** Muscle mass depletion, overhydration, and inflammatory state have been related to impaired physical function in chronic kidney disease patients. The relationship between bioelectrical impedance analysis (BIA) parameters, such as hydration status and phase angle (PhA), with physical function in peritoneal dialysis (PD), is still not well-established. Therefore, the objective was to evaluate the association of BIA parameters (overhydration index and PhA) and inflammatory markers with physical function in patients on PD.

**Methods:** The present cross-sectional study enrolled PD patients. Multifrequency BIA was performed to obtain overhydration index and PhA. The Short Physical Performance Battery (SPPB) test battery was applied to assess physical function. The time to complete the 4-m gait test and sit-to-stand test was also considered for physical function assessment. The inflammatory markers tumor necrosis factor-alpha and C-reactive protein levels were determined. Multiple linear regression models were performed, with the physical function variables as dependent variables, adjusted for age, diabetes, and sex.

**Results:** Forty-nine PD patients were enrolled, 53.1% (*n* = 26) women; mean age, 55.5 ± 16.3 years. There were significant correlations between PhA and SPPB (*r* = 0.550, *p* < 0.001), time of 4-m gait test (*r* = −0.613, *p* < 0.001) and sit-to-stand test and (*r* = −0.547, *p* < 0.001). Overhydration index was significantly correlated with SPPB, 4-m gait test (*r* = 0.339, *p* = 0.017), and sit-to-stand test (*r* = 0.335, *p* = 0.019). Inflammatory markers were not significantly correlated with physical function parameters. In the multiple linear regression analysis, PhA was associated with physical function parameters, even after adjustments. Overhydration index was associated with all physical function tests only in the models with no adjustments.

**Conclusion:** PhA was independently associated with physical function in PD patients. Inflammatory markers and overhydration index were not associated with physical function.

## Introduction

Bioelectrical impedance analysis (BIA) has been used in clinical practice to assess patients with chronic kidney disease (CKD) on dialysis. BIA provides raw data regarding electrical current conductivity through the body tissues, such as resistance and reactance. The relation among these parameters may be converted into physiological parameters related to body composition and hydration status (1).

BIA was recommended by the National Kidney Foundation–Kidney Disease Outcomes Quality Initiative in the Nutrition Guideline in CKD, as a method capable of assessing body composition in patients on hemodialysis. However, there is insufficient evidence to suggest using BIA to assess body composition in peritoneal dialysis (PD) patients (2).

Regarding hydration status evaluation, one of the parameters obtained by BIA is the overhydration index (OH), which assesses fluid overload by the difference between measured and expected extracellular water in normal situations (3). OH is considered an important predictor of the evolution of patients on dialysis, as it helps with blood pressure control (4). Moreover, OH has been associated with lower survival in both hemodialysis and PD patients (5, 6).

Phase angle (PhA), a measure of the relationship between resistance and reactance, reflects cell membranes integrity, cellularity, and cell function (7). PhA has been related to hydration status (8), nutritional status (9), and mortality risk (10) in PD patients.

CKD is characterized by metabolic and hormonal disturbances, which culminates in skeletal muscle wasting and disfunction. Decreased physical function has been associated with poor outcomes, such as disability, falls, fracture, hospitalization, and mortality (11). In dialysis and non-dialysis CKD patients, muscle mass depletion, overhydration, and inflammatory state have been related to impaired physical function (12, 13).

However, the relationship between BIA parameters, such as hydration status and PhA, with physical function in PD is still not well-established. Therefore, the objective of the study is to evaluate the association of BIA parameters (OH and PhA) and inflammatory markers with physical function in patients with CKD on PD.

## Methods

This was a secondary analysis of data from a study previously published (14), which enrolled patients aged 18 years or above, with CKD on PD (for at least 3 months) at the Dialysis Unit from the Clinics Hospital of Botucatu Medical School (Botucatu, Brazil). The protocol was approved by the Ethics and Research Committee (CAAE 61634816.4.0000.5411), and the participants gave written informed consent.

Clinical, demographic data, and routine laboratory tests were collected from medical records (sex, age, comorbidities, underlying disease, PD modality, Kt/V, serum hemoglobin, creatinine, urea, albumin, C-reactive protein).

### Inflammatory Markers

Blood collection was performed in the morning, after PD. C-reactive protein levels were determined by immunoassay (Vitros, Johnson and Johnson Clinical Diagnostics, Rochester, NY) at the specialized chemistry laboratory of the Hospital of Botucatu Medical School. Blood samples were centrifuged, aliquoted, and kept frozen at −80°C for tumor necrosis factor-alpha (TNF-α) levels determination, using an enzyme-linked immunosorbent assay according to the manufacturer's instructions (R&D System, Inc., Minneapolis, USA).

### Bioelectrical Impedance Analysis

All the patients underwent anthropometric and bioimpedance assessment with no dialysate in the peritoneal cavity in the morning, after PD. Measurements of body weight, height, and calculation of body mass index were carried out.

Multifrequency BIA (Body Composition Monitor, BCM—Fresenius Medical Care®, Germany) was performed with the patients in the recumbent position and all metal accessories removed. The BCM measures body resistance and reactance to electrical currents of 50 discrete frequencies, ranging from 5 to 1,000 kHz. Values of OH and PhA in the frequency of 50 kHz were considered. The OH was obtained by the BCM device, considering the difference between the measured extracellular water (ECW) and the predicted values based on fixed hydration on lean and adipose tissue mass.

### Physical Function Assessment

To assess physical function, the Short Physical Performance Battery (SPPB) was used. This assessment was performed at the same day of BIA, after PD. SPPB is composed of a set of three tests: balance, a 4-m gait and sit-to-stand tests. These tests are useful to predict the performance of the lower limbs. The scores were assigned to each test, ranging from zero to four (15, 16).

Besides the SPPB total score, the time to complete the 4-m gait test and sit-to-stand test was considered for physical function assessment.

### Statistical Analysis

The Kolmogorov-Smirnov test was performed to assess the normality of the data. Data were expressed as mean and standard deviation, median, and interquartile variation, according to the distribution. Frequencies were expressed as percentages.

Comparisons between male and female, and between diabetic and non-diabetic patients were performed using Student's *t* or Mann–Whitney tests.

The patients were grouped according to OH and PhA tertiles. Tertiles were compared, using one-way ANOVA with Tukey *post-hoc* or Kruskal–Wallis with Dunn *post-hoc* test.

Correlations between physical function parameters and other variables were assessed. For this, Pearson and Spearman coefficient correlations were used.

Multiple linear regression models were performed, with the physical function variables as dependent variables, adjusted for age, diabetes, and sex.

The analyses were performed, using IBM SPSS Statistics V22.0 (IBM Corp., Armonk, NY, USA). The level of significance was set at *p* < 0.05.

## Results

Forty-nine PD patients were enrolled, with mean age 55.5 ± 16.3 years, 53.1% (*n* = 26) female. The patients in different modalities of PD were included, 61.2% (*n* = 30) in continuous cyclic PD, 34.7% (*n* = 17) in night intermittent PD, and 4.1% (*n* = 2) in continuous ambulatory PD. The most prevalent comorbidities were arterial hypertension (79.6%, *n* = 39) and diabetes (26.5%, *n* = 13). The main underlying diseases were glomerulonephritis, 18.4% (*n* = 9); diabetic nephropathy, 16.3% (*n* = 8); hypertensive nephropathy, 16.3% (*n* = 8); undetermined, 24.5% (*n* = 12); and other causes, 24.5% (*n* = 12). Clinical and nutritional characteristics of PD patients are shown in Table 1.

**Table 1:** Clinical and nutritional characteristics of PD patients.

**Variables**	**Total (*n* = 49)**
Dialysis vintage (months)	10.0 (5.0–18.0)
Kt/V total^*^	2.2 ± 0.5
Kt/V peritoneal^**^	1.4 (1.1–1.7)
Kt/V renal^**^	0.6 (0.3–1.1)
Weight (kg)	68.8 ± 15.5
Body Mass Index (Kg/m^2^)	25.8 ± 4.3
Phase angle (°)	5.4 ± 1.1
Overhydration (L)	0.8 ± 1.1
Hemoglobin (mg/dL)	11.3 ± 1.5
Urea (mg/dL)	99.3 ± 24.9
Creatinine (mg/dL)	9.1 ± 2.8
Albumin (g/dL)	3.6 ± 0.4
C-reactive protein (mg/L)	0.5 (0.5–1.0)
Tumor necrosisfactor-alpha (ng/L)	16.9 (13.8–21.4)

*Data are expressed as numbers only, mean ± standard deviation, number (%), or median (interquartile range)*.

*Kt/V: Fractional clearance of urea;*

* *n = 47;*

** *n = 46*.

Results of physical function assessments of both genders were compared. There were no differences between male and female (SPPB: *p* = 0.324, gait test: *p* = 0.109 and sit-to-stand test: *p* = 0.248). In the comparison between the patients with diabetes or not, there were differences regarding age (*p* = 0.003), SPPB (*p* = 0.025) and sit-to-stand test (*p* = 0.009). OH (*p* = 0.184), PhA (*p* = 0.193), CRP (*p* = 0.572), TNF-α (*p* = 0.937), and gait test (*p* = 0.150) did not differ between diabetic and non-diabetic patients.

Forty-seven patients (96.0%) reached the maximum score in the balance test. The results of physical function assessments and inflammatory markers are shown in Table 2, as well as the comparison among OH tertiles. OH ranged from −1.0 to 3.2 L. PhA ranged from 3.2° to 7.8°. The comparison among PhA tertiles is shown on Table 3.

**Table 2:** Comparison of different parameters among overhydration tertiles in patients on peritoneal dialysis (*n* = 49).

**Variables**	**1st Tertile OH <0.27 (*n* = 17)**	**2nd Tertile OH ≥0.27 to <1.26 (*n* = 16)**	**3rd Tertile OH ≥ 1.26 (*n* = 16)**	***p***
Age (years)	52.4 ± 14.4	52.4 ± 16.8	61.8 ± 16.8	0.172
Female sex [*n* (%)]	10 (58.8)	8 (50.0)	8 (50.0)	0.841
PD modality [*n* (%)]				0.862
CAPD	1 (5.9)	0	1 (6.2)	
CCPD	10 (58.8)	11 (68.8)	9 (56.3)	
NIPD	6 (35.3)	5 (31.2)	6 (37.5)	
Short physical performance battery (points)	10.0 (9.0–11.0)	9.5 (8.0–10.8)	8.5 (7.0–9.8)^a^	0.035
Gait test (seconds)	4.21 (3.5–5.3)	4.4 (4.1–5.2)	5.0 (4.5–7.1)^a^	0.029
Sit-to-stand test (seconds)	15.2 ± 3.6	15.8 ± 3.9	19.5 ± 4.7^a^^b^	0.009
C-reactive protein (mg/L)	0.5 (0.5–1.3)	0.5 (0.5–0.6)	0.6 (0.5–1.4)	0.387
Tumor necrosisfactor-alpha (ng/L)	16.94 (14.54–23.23)	17.4 (11.6–21.3)	15.5 (13.7–20.0)	0.527

*Data are expressed as numbers only, mean ± standard deviation, number (%), or median (interquartile range). Comparison using one-way ANOVA with the Tukey post-hoc test or Kruskal–Wallis with Dunn post-hoc*.

a *p < 0.05 when compared with first tertile;*

b *p < 0.05 when compared with second tertile*.

**Table 3:** Comparison of different parameters among phase angle tertiles in patients on peritoneal dialysis (*n* = 49).

**Variables**	**1st Tertile PhA <4.92 (*n* = 16)**	**2nd Tertile PhA ≥4.92 to ≤5.87 (*n* = 17)**	**3rd Tertile PhA >5.87 (*n* = 16)**	***p***
Age (years)	67.8 ± 12.0	54.9 ± 13.2^a^	43.7 ± 14.4^a^^b^	0.000
Female sex [*n* (%)]	10 (62.5)	11 (64.7)	5 (31.2)	0.103
PD modality [*n* (%)]				0.622
CAPD	1 (6.2)	1 (5.9)	0	
CCPD	8 (50.0)	10 (58.8)	12 (75.0)	
NIPD	7 (43.8)	6 (35.3)	4 (25.0)	
Short physical performance battery (points)	8 (7.0–9.0)	10 (9.0–11.0)^a^	10 (9.2–11.0)^a^	0.002
Gait test (seconds)	6.2 (4.8–7.0)	4.5 (4.0–5.1)^a^	4.2 (3.6–4.5)^a^	0.000
Sit-to-stand test (seconds)	20.0 ± 4.4	16.0 ± 4.4^a^	14.5 ± 2.2^a^	0.001
C-reactive protein (mg/L)	0.5 (0.5–1.6)	0.5 (0.5–1.3)	0.5 (0.5–0.7)	0.412
Tumor necrosisfactor-alpha (ng/L)	16.7 (14.2–20.9)	14.5 (11.1–17.4)	19.8 (15.6–23.2)	0.058

*Data are expressed as numbers only, mean ± standard deviation, number (%), or median (interquartile range). Comparison using one-way ANOVA with the Tukey post-hoc test or Kruskal–Wallis post-hoc*.

a *p < 0.05 when compared with first tertile;*

b *p < 0.05 when compared with second tertile*.

In the correlation analysis, there was a positive correlation between PhA and SPPB and negative correlations of PhA with time of a 4-m gait and sit-to-stand tests. OH was negatively correlated with the total SPPB score, and positively correlated with a 4-m gait and sit-to-stand tests. Inflammatory markers were not significantly correlated with physical function parameters (Table 4). Dialysis vintage, renal Kt/V, BMI, urea, and hemoglobin were not significantly correlated with physical function tests. Albumin was correlated only with the gait test (*r* = −0.400, *p* = 0.004). Creatinine was correlated with all the physical function test (SPPB *r* = 0.389, *p* = 0.006; gait test *r* = −0.430, *p* = 0.002; sit-to-stand *r* = −0.379, *p* = 0.007).

**Table 4:** Correlations between bioimpedance parameters and physical function tests in patients on peritoneal dialysis (*n* = 49).

**Variables**	**Phase angle (**^****°****^**)**		**OH (L)**		**CRP (mg/L)**		**TNF-α** **(ng/L)**	
	***r***	***p***	***r***	***p***	***r***	***p***	***r***	***p***
Short physical performance battery (points)	0.550	0.000	−0.316	0.027	−0.013	0.931	0.110	0.450
4-meter gait test (seconds)	−0.613	0.000	0.339	0.017	0.136	0.350	−0.169	0.245
Sit-to-stand test (seconds)	−0.547^*^	0.000	0.335^*^	0.019	0.020	0.894	−0.131	0.368

*OH, overhydration; CRP, C-reactive protein; TNF-α, Tumor necrosis factor-alpha*.

* *Pearson coefficient correlation. Coefficients with no sign refer to Spearman coefficient correlation*.

Multiple linear regression models were performed with the SPPB, gait test, and sit-to-stand test as dependent variables (Table 5). Age and diabetes were chosen as adjustments due to the association with physical function in univariate analysis. Sex was also included as an adjustment. Although serum creatinine was correlated with physical function tests, in multiple linear regression models, creatinine did not improve or change the results. Therefore, as the sample size is small, we did not add creatinine to the final models.

**Table 5:** Multiple linear regression models with short physical performance battery, the gait test, and the sit-to-stand test as dependent variables in patients on peritoneal dialysis (*n* = 49).

**Dependent variable**	**Independent variable**	**Model**	**β**	**CI 95%**	***p***	**Adjusted R^2^**
Short physical performance battery (points)	Phase angle (°)	1	0.544	0.474–1.261	<0.001	0.281
		2	0.324	0.058–0.977	0.028	0.359
		3	0.340	0.085–1.000	0.021	0.370
		4	0.325	0.008–1.028	0.047	0.356
	Overhydration (L)	1	−0.306	−0.913 to −0.042	0.032	0.075
		2	−0.208	−0.701–0.053	0.090	0.331
		3	−0.194	−0.684–0.079	0.117	0.328
		4	−0.206	−0.701–0.059	0.095	0.339
Gait test (seconds)	Phase angle (°)	1	−0.585	−1.439 to −0.607	<0.001	0.329
		2	−0.356	−1.102 to −0.142	0.012	0.417
		3	−0.369	−1.125 to −0.166	0.009	0.424
		4	−0.293	−1.038–0.014	0.056	0.430
	Overhydration (L)	1	0.289	0.015–0.974	0.044	0.064
		2	0.184	−0.089–0.716	0.124	0.365
		3	0.172	−0.114–0.701	0.154	0.359
		4	0.192	−0.063–0.717	0.098	0.418
Sit-to-stand test (seconds)	Phase angle (°)	1	−0.547	−3.226 to −1.228	<0.001	0.285
		2	−0.372	−2.712 to −0.315	0.014	0.329
		3	−0.391	−2.774 to −0.410	0.009	0.353
		4	−0.373	−2.836 to −0.199	0.025	0.339
	Overhydration (L)	1	0.335	0.231–2.429	0.019	0.093
		2	0.246	−0.007–1.965	0.052	0.296
		3	0.229	−0.084–1.901	0.072	0.300
		4	0.242	−0.019–1.945	0.054	0.319

*Model 1: no adjustments; Model 2: adjusted by age; Model 3: adjusted by age and diabetes; Model 4: adjusted by age, diabetes, and sex*.

In the multiple linear regression final models, OH was associated with all physical function tests only in the models with no adjustments. PhA was independently associated with all physical function tests after adjustments, except with the gait test when diabetes was included as an adjustment.

## Discussion

Although BIA has been used in the last decades for body composition assessment, it is not a direct method for this evaluation (7). BIA is based on electrical properties of tissues, and the body compartments are estimated from equations that include raw impedance parameters and anthropometric measurements (17).

More recently, the use of raw and associated impedance parameters has gained attention. Kidney failure patients are characterized by fluid imbalance, which turn BIA even more valuable. Huang et al. showed that age, diabetes, and fluid overload were independently associated with lower PhA in PD patients. Moreover, in the same study, lower PhA was associated with higher cardiovascular and all-cause mortality (10). Increased OH values were associated with mortality risk, as evidenced in a systematic review, enrolling studies with kidney failure patients (18).

The present study aimed to evaluate the association of BIA parameters (PhA and OH), and inflammation with physical function in PD patients. PhA was positively correlated with the SPPB total score and negatively correlated with the time to complete a 4-m gait and sit-to-stand tests. As expected, these results show the association of increased PhA with better physical function.

In addition to the association of PhA with better physical function, OH was negatively correlated with the SPPB total score and positively correlated with the time to complete the 4-m gait and sit-to-stand tests. The patients in the third tertile had worse physical function compared with the first tertile. However, OH was not independently associated with physical function after adjustments in multiple analyses.

Inflammation is considered one of the aspects that lead to decrease of physical function (19, 20). Since overhydration status may increase inflammation in PD patients (21), we expected an association of both inflammatory markers and OH with physical function. However, the inflammatory markers considered in our study were not associated with worse physical function, as well as OH.

In the hemodialysis patients, the presence of fluid overload can slow the gait speed. The fluid overload significantly impacts on the physical performance of the patients over time, as observed in a longitudinal study by Carlos et al. (12). On the other hand, excessive fluid withdrawal in hemodialysis sessions can lead to intradialytic hypotension, which is associated with a reduced gait after a dialysis session (22, 23).

In PD, data about the impact of increased fluid on the physical function assessed by walking are scarce. A walking variable may be reduced in these patients due to conditions such as physical inactivity, peripheral neuropathy, and muscle strength (24). However, such variables were not evaluated in the present study, which may be an explanation for the lack of association of physical function with OH in the regression model.

In the multiple linear regression analyses, the association of PhA with the sit-to-stand test and SPPB was maintained even after adjusting for age, diabetes, and sex. All the physical function tests used in the present study reflect common daily living activities, i.e., getting up from a chair or walking small distances. Moreover, these tests are easy, fast, portable, and inexpensive procedures to measure physical performance.

PhA has been associated with physical function in many conditions. Recently, Vincenzo et al. (25) have showed in a metanalysis that PhA is decreased in sarcopenic subjects. Kosoku et al. (26) found a negative correlation between PhA and sarcopenia in kidney transplant recipients. Sarcopenia is defined as the presence of both muscle mass and physical function decrease (27).

Although the relationship between PhA and physical function in PD is still not well-established, there are studies associating PhA with muscle function in other CKD populations. Muscle function, assessed by handgrip strength, was associated with PhA in maintenance hemodialysis (28, 29) and kidney transplant recipients (30). Moreover, in the hemodialysis patients, PhA was associated with exercise tolerance, which was assessed by a 6-min step test, and peak torque of knee extensors (28).

A possible explanation for the relationship between PhA and physical function is based on cellular structures and its interaction with the electrical current. The structure and the function of cells are dependent on cell membranes, which are composed of electrically non-conducting lipid bilayers with associated proteins (31). The protein–lipid–protein sandwiched structure provides a reactance to the applied alternating current (32). The reactance arises from cell membranes, while resistance from extra- and intracellular fluid (17). The impedance is the result of two vectors representing resistance and reactance. PhA is the angle between impedance and resistance. Therefore, the higher the reactance, the larger the PhA will be for a given resistance (33). In other words, the higher PhA, the better cell membrane structure, cell mass, cellular integrity, and cell function.

The main limitation of this study is the single-center small sample size, which does not allow the inclusion of many variables in the multiple analyses. However, due to the scarcity of studies assessing the association of PhA and OH with physical function in PD, the results may be significant for PD patients. Thus, studies with a larger number of patients are still necessary.

In conclusion, bioimpedance parameters (PhA and OH) were correlated with physical function tests, but only PhA was independently associated with physical function tests in the PD patients. Inflammatory markers were not associated with physical function in this sample. In this sense, easy-to-apply tools with good accuracy are important to identify the risk of reduced physical function, allowing interventions to avoid worse outcomes.

## Data Availability Statement

The raw data supporting the conclusions of this article will be made available by the authors, without undue reservation.

## Ethics Statement

The studies involving human participants were reviewed and approved by Research Ethics Committee of the Faculty of Medicine of Botucatu-Universidade Estadual Paulista (UNESP). The patients/participants provided their written informed consent to participate in this study.

## Author Contributions

VMS, MZCS, BPV, and JCTC were responsible for the research idea and the study design. VMS, MZCS, NSCR, MSD, and FLC performed the data acquisition. VMS, MZCS, and BPV performed the data analysis and the interpretation, were involved in the statistical analysis, and drafted the manuscript. BPV, JCTC, and MFM were responsible for supervision and mentorship. All the authors provided intellectual content to the work and gave final approval of the version to be published.

## Conflict of Interest

The authors declare that the research was conducted in the absence of any commercial or financial relationships that could be construed as a potential conflict of interest.
